# Telehealth Adoption in an Outpatient Oncology Ward: A Best Practice Implementation Project

**DOI:** 10.3390/nursrep12030050

**Published:** 2022-07-13

**Authors:** Filipa Ventura, Helena Domingues, Gisela Almeida, Daniela Cardoso, Rogério Rodrigues, Isabel Moreira, Mariana Pires, Inês Gomes, Rosa Silva, Cláudia Oliveira, Ana Filipa Cardoso, Liliana Ribeiro, Cristina Costeira

**Affiliations:** 1 The Health Sciences Research Unit: Nursing (UICISA:E), Nursing School of Coimbra (ESEnfC), 3000 Coimbra, Portugal; dcardoso@esenfc.pt (D.C.); rogerio@esenfc.pt (R.R.); imoreira@esenfc.pt (I.M.); rosacgsilva@esenfc.pt (R.S.); claudia.oliveira@ipiaget.pt (C.O.); cardoso.anafilipa@gmail.com (A.F.C.); lianaescada@gmail.com (L.R.); cristina.costeira@ipleiria.pt (C.C.); 2 Portuguese Institute of Oncology of Coimbra Francisco Gentil, E.P.E. (IPO Coimbra), Outpatient Oncology Ward, 3000 Coimbra, Portugal; 3325@ipocoimbra.min-saude.pt (H.D.); 3408@ipocoimbra.min-saude.pt (G.A.); 3896@ipocoimbra.min-saude.pt (M.P.); 3952@ipocoimbra.min-saude.pt (I.G.); 3 Portugal Centre for Evidence Based Practice: A JBI Centre of Excellence (PCEBP/JBI), 3000 Coimbra, Portugal; 4 Research in Education and Community Intervention (RECI), Jean Piaget School of Health, 8300 Silves, Portugal; 5 Center for Innovative Care and Health Technology (ciTechcare), School of Health Sciences, Polytechnic of Leiria, 2411 Leiria, Portugal

**Keywords:** telehealth, outpatient oncology, implementation science, best practices, information and communication technology

## Abstract

Telehealth is increasingly taking place to support the transition of care and self-management of people living with cancer in outpatient oncology settings. Despite its recognised value, the scientific evidence points to disparities with regard to implementation of telehealth that might compromise the equity of access. Following the Joanna Briggs Institute (JBI) implementation approach, this project aims to promote the implementation of best practice recommendations for telehealth adoption in an outpatient oncology setting. Assisted by the Practical Application of Clinical Evidence System (PACES), the implementation process comprises three phases of (i) a baseline audit, (ii) feedback to the healthcare team and establishment of implementation strategies with the Getting Research into Practice (GRiP) tool, and (iii) a follow-up audit. The project is expected to allow the identification of barriers and facilitators for the implementation of telehealth in outpatient oncology and develop a strategy plan for its adoption, with the involvement of end-users and stakeholders. The successful adoption of telehealth according to the best available evidence will likely enhance equity of access to healthcare and quality of care at a distance.

## 1. Introduction

The advances in cancer diagnosis and treatment have made it possible to shift most of treatment trajectories from inpatient to the outpatient setting [1]. Along with the need to reorganise oncological care and treatment to meet the supportive needs of people living with cancer at a distance, telehealth has been increasingly receiving attention and put in place to complement the communication processes in outpatient oncology care [2].

Telehealth was set as a global health priority for implementation by the World Health Organization (WHO) already in 2005 [3]. Generally, it can be defined as the use of information and communication technology to provide healthcare services in real-time or asynchronously [4]. It might be used as a resource to communicate with patients and their families (i.e., teleconsultation, teletriage), or to conduct multidisciplinary case discussions with or without the patient’s involvement. Digital health interventions for support at a distance through mobile applications and electronic health records are two other features of telehealth [2].

Particularly in cancer care, telehealth allows the provision of self-management support, telemonitoring, and health education and has become essential in the daily life of the person with oncological disease [5]. As complementary resources to healthcare, telehealth interventions through mobile applications have shown evidence of improving person-relevant outcomes, such as self-efficacy and healthcare participation, as well as patient-reported outcomes such as depression, anxiety, pain, fatigue, and wellbeing [2]. Digital health interventions are generally well accepted by users and scientific evidence reveals their impact in reducing emergency services [6].

Even though the telehealth phenomenon is not new, its adoption was sometimes controversial and subject to uncertainties from the perspectives of both healthcare professionals and patients. The forced need to minimise travelling and face-to-face contact brought on by the COVID-19 pandemic contributed greatly to the rapid adoption of telehealth solutions, reinforcing the need for healthcare models with integrated supportive care at a distance [7]. Along with the spread of telehealth, many myths were overcome, yet some challenges remain.

Despite the recommendations for cancer management [8], significant disparities were observed in relation to the adoption of telehealth interventions during the COVID-19 pandemic. These were related to the patient’s geographical location (i.e., urban vs. rural), culture, language proficiency, comorbidities, and socio-demographic elements (e.g., age, digital literacy, marital status, gender) [9,10]. Particularly concerning geographical location, telehealth allows the overcoming of transportation barriers. On the other hand, rural areas that are more likely to experience transportation barriers are also more likely to present difficulties with regard to the adoption of telehealth due to lack of support on-site [11].

More recently, standards were provided by the American Society of Clinical Oncology and reinforced by the European Society for Medical Oncology that aim to establish recommendations for various telehealth domains. These standards were derived from a systematic search for studies covering the main telehealth questions, which were then synthesised and revised by an expert panel for consensus and guidance [12]. Importantly, the use of telehealth involves more than just having the technology in place [13]. The organisational structure, the clinical workflow, the multidisciplinary healthcare team, and the patient and their family need to be considered and involved to address barriers to acceptance and equal access to telehealth [14].

Particularly regarding digital health interventions, challenges are found at the development and implementation phases. The concerns refer to the suitability of these resources for most of the patients and their compliance with the intervention, as well as the intervention’s transferability across healthcare systems and living environments [15]. This knowledge resulting from recent clinical studies reinforces the transferability issue already identified in the expansion years of eHealth, where the implementation of interventions assisted by technology was hampered in routine clinical practice, despite their recognised effectiveness [16]. The need for high-quality research with systematic and effective strategies to improve patient and healthcare professionals’ engagement in the design, delivery, and implementation of telehealth interventions is still on today’s agenda for supportive care through telehealth [2].

Altogether, the scientific evidence points to the importance of exploring the optimal implementation strategy, along with identifying barriers and facilitators of telehealth adoption [17]. Considering the evidence-to-practice gap aligned with the “research waste” phenomenon [18], research endeavours have been deployed towards improving the acceptability and clinical relevance of health interventions, including those assisted by digital technology [2]. Consequently, scholars have rendered theories, models, and frameworks to enable assessment and management of complex elements [19]. This study adopts the lens of complexity theory applied to health and care technologies as health interventions according to the Nonadoption, Abandonment, Scale-up, Spread, and Sustainability framework (NASSSf). The NASSSf supports researchers to predict and evaluate the success of a technology-mediated healthcare program. The framework enables researchers to pose questions to several domains and to the interaction and mutual adoption between these domains over time, while raising the challenges pertaining to each of the domains. The more domains are considered complex, the harder it is for an intervention to become mainstream in clinical practice [20].

Accordingly, the primary objective of this study is to promote the implementation of best practice recommendations for telehealth adoption in an outpatient oncology setting by answering the main research question: what is the best practice for the adoption of telehealth by both patients and healthcare professionals in outpatient oncology?

The study-specific objectives are stated as follows:

1. Determine the compliance of current practice with the evidence-based criteria before and after implementation;
2. Identify barriers and facilitators to improve compliance with the established best-practice criteria;
3. Develop and implement strategies to address noncompliance practice domains;
4. Assess the acceptance and readiness for telehealth adoption by end-users and stakeholders.

## 2. Materials and Methods

The Joanna Briggs Institute (JBI) approach to evidence implementation guides the current project [21]. The methodology will be applied by means of the Practical Application of Clinical Evidence System (JBI-PACES) over a nine-month period, with the expected conclusion in December 2022. It entails seven sets of activities that can be usefully organised in three project phases: (i) stakeholder engagement and baseline audit, (ii) feedback and discussion of implementation strategies, (iii) follow-up audit and sustainability.

### 2.1. Setting

This evidence implementation project will be deployed at the outpatient oncology department of an oncology hospital located in the Central Region of Portugal. The outpatient oncology ward is in an urban location in the Central Region of Portugal, where patients come with a referral from their family doctor, other hospitals, or private clinics in their area of residence to receive assistance with cancer diagnosis and treatment. The outpatient oncology ward runs from 9 am–8 pm on weekdays, receiving a daily average of 100 people for antineoplastic treatment (e.g., chemotherapy, immunotherapy). Patients attending the ward are adults aged 18 years old or older. Oncological diagnoses vary and include head and neck, dermatological, digestive, endocrinological, gynaecological, haematological, breast, bone and soft tissue, pulmonary, neuroendocrine, urological, and central nervous system diagnoses.

Healthcare is provided by a multidisciplinary team that is composed of a nursing team of 15 nurses and the head of nursing, the medical oncology team of 11 intern physicians, 19 oncologists, and the clinical director on a permanent basis. Other healthcare professionals (e.g., pharmacists, psycho-oncologists, physiotherapists, nutritionists) are called upon to intervene by referral from the resident multidisciplinary team. In addition to the open-space treatment room equipped with 22 armchairs and 6 beds, the outpatient oncology ward also has an office for unscheduled consultations, and an office for an oncologist resident as a support to the outpatient oncology ward.

### 2.2. Sample

In a cross-sectional study approach, with a convenience sampling technique, users receiving antineoplastic therapy on the days of the baseline and follow-up audits will be approached by the research team to participate in a structured interview according to the evidence criteria checklist. An audit period of one week is foreseen between 1:00 pm and 4:30 pm. Considering the evidence on the sample size adequacy of other implementation studies that followed the JBI approach, each audit is estimated to have a minimum sample size of 30 patients.

All the patients attending the outpatient oncology ward for the purpose of receiving antineoplastic treatment will be asked to participate in the study by a member of the research team. Inclusion criteria are defined regardless of age, gender, geographical residential area, cancer diagnosis, or antineoplastic treatment. Patients who are receiving their first antineoplastic treatment session will be excluded from participation as they are in a vulnerable situation and do not have experience with the potential need for telehealth interventions. Patients who lack the ability to understand and answer the questions will also be excluded from participation.

The medical and nursing records in the patient’s electronic journal will also be analysed against the evidence criteria checklist for those patients that consent to participate in the audit interviews. This procedure is intended to allow triangulation of the compliance assessment.

In relation to health professionals, all the healthcare professionals (i.e., doctors and nurses) at the outpatient oncology ward will be invited to participate, excluding the members who will participate as part of the evidence implementation team, and those who do not provide healthcare or are on leave (e.g., sick leave, vacation, parental leave). Healthcare professionals who meet the inclusion criteria will be approached by the implementation team to answer a questionnaire (online or in person) according to the evidence criteria checklist.

### 2.3. Ethical Consideration

The project was reviewed by the ethics board of the Portuguese Institute of Oncology of Coimbra and received ethical approval.

### 2.4. Phase I: Stakeholder Engagement and Baseline Audit

The implementation project will be carried out in collaboration with stakeholders at the organisation’s macro-level (e.g., the clinical director and head of nursing), meso-level (e.g., the manager of the quality improvement office), and micro-level (the clinical director and head of nursing of the outpatient oncology ward).

The implementation team will be composed of scholars and researchers in healthcare sciences and experts in implementation science, representatives of healthcare professionals from the outpatient oncology ward (i.e., two medical oncology physicians and two specialist nurses), and patient representatives.

#### Audit Criteria

An evidence summary was developed by the JBI to address the following question: what is the best available evidence regarding the adoption of telehealth interventions for adult cancer patients receiving cancer treatment?

The JBI evidence summary [22] reviewed and synthesised six studies of high-quality evidence in seven best practice recommendations:

1. Healthcare professionals should consider how telehealth may impact the patient–professional relationship and when face-to-face visits may be more appropriate for each individual patient.
2. Healthcare organisations should have policies and procedures detailing risk management, ethical considerations including consent and privacy and legal issues, and technical considerations for telehealth in cancer patients.
3. If possible, healthcare organisations should provide patients with a device to participate in telehealth interventions.
4. Healthcare professionals should receive training in the use of the applications and devices used for telehealth.
5. Healthcare professionals should identify and address potential barriers to telehealth use for each patient.
6. Healthcare organisations should outline costs to patients (if any) for telehealth consultations.
7. A dedicated technological support person should be available to troubleshoot.

The best practice recommendations were operationalised into an evidence criteria checklist to allow for audit evaluation at baseline and follow-up. Each audit criterion will be assessed through various sources to allow for a triangulated assessment. Specifically, criteria 2, 4, 5, and 6 will be assessed through inquiry of the patients attending the outpatient oncology ward and their medical electronic journals. Criterion 3 will be assessed through inquiry of the healthcare professionals of the multidisciplinary team at the outpatient oncology ward and the audited patients’ electronic medical journals. Full compliance (i.e., 100%) is determined if the assessment of both data sources is positive (Table 1).

In addition to the audit criteria, both patients and healthcare professionals will be questioned about their perspectives on telehealth. Specifically, they will be asked about their perceived barriers, facilitators, advantages, and disadvantages to the adoption of telehealth, as well as its adequacy for specific situations.

### 2.5. Phase II: Feedback and Design and Implementation of Strategies

The results of the baseline audit will be presented and discussed among the implementation team and further enhanced by the feedback of the organisation’s multilevel stakeholders. Feedback on this process will be given and discussed with the healthcare professionals to promote their engagement in the implementation process and enhance their awareness of potential barriers. In addition to a joint presentation, individual dissemination strategies will be used to provide the results from the baseline audit.

The Getting Research into Practice tool (GRiP) will be used to assist in the identification of barriers and facilitators for the implementation of the best practice recommendations according to the JBI evidence summary, as well as in identifying the strategies to overcome the latter. According to the JBI evidence implementation approach, the GRiP method enables the comparison of the audit results, and the identification of barriers and facilitators for the implementation of the evidence in telehealth adoption. GRiP will assist in the co-development of the implementation strategies to reduce the gap between the scientific evidence and the clinical practice [21].

### 2.6. Phase III: Follow-Up Audit

Aiming to determine the efficacy of the implemented strategies in improving compliance with the evidence criteria, a follow-up audit will be conducted in Phase III. During this phase, a sustainability plan will be discussed and designed by the implementation team to ensure the continuity and update of the implantation strategies, and future issues to be addressed will be identified.

## 3. Expected Results and Discussion

Similar to other studies where evidence implementation was guided by the JBI evidence implementation approach, compliance rates to each criterion are expected to increase from the baseline to the follow-up audit.

As highlighted by the scientific evidence, telehealth has great potential to bridge communication and support gaps in healthcare, with a positive impact on both personal, clinical, and organisational outcomes. Yet, the inequity of access to telehealth interventions is still an issue. Thorough and systematic research work that enables the implementation of best practices in the clinical workflow might contribute to overcoming equity barriers [2].

Delivery of telehealth requires more than just technological devices. Previous barriers to telehealth have included the need to change work processes [13], which points to the engagement of organisational multilevel stakeholders as essential. Particularly concerning the integration of telehealth interventions in the existing clinical workflow, evidence from the COVID-19 pandemic period highlights the impact of the overuse of telehealth interventions on the patient–physician relationship. If aiming to achieve the full potential of telehealth interventions, it is important to respect patient preferences and assure a balance between telehealth interventions and in-person consultations [23,24].

Additionally, scholars point out the risks of exacerbating the digital divide [13], as also highlighted previously with regard to the disparities of access depending on various socio-demographic and clinical characteristics [9,10]. Even though the sampling of patients will occur consecutively, older adults, persons with lower digital literacy, or people with worse wellbeing are more likely to be less interested in enrolling in the study a priori. This aspect might hinder a comprehensive assessment of the phenomenon of telehealth adoption.

Another issue that might compromise telehealth’s full potential is the lack of trust of both patients and healthcare professionals in telehealth interventions. The possibility to overcome the digital divide and gain users’ trust resides partially in the ease of use of applications, and their reliability and flexibility to adapt across systems. Moreover, patients using telehealth interventions might have the sense of being over-observed, which might be experienced as a threat to their privacy and confidentiality. Such experiences might lead them to the nonadoption of telehealth. Therefore, it is important to promote the discussion of privacy plans that suit everyone’s preferences, as they will be valued differently depending on the person’s culture and beliefs [13].

The perspective of healthcare professionals cannot be neglected either. Evidence shows contradictory views on the advantages of telehealth with regard to clinical effectiveness and enhanced access to care [14]. Furthermore, other studies have identified that barriers related to technological difficulties and low digital literacy might be equally present in the healthcare team [17]. The engagement of the healthcare team in the identification of barriers and facilitators for telehealth implementation is therefore crucial to enable its acceptance and usability.

Finally, additional efforts involve the improvement of affordability of telehealth solutions, with the demand for technological devices and good Internet communication. To that end, funding strategies might need to be revised and financial incentives for healthcare organisations might be of value to enhance sustainability of care pathways with integrated telehealth [13].

In conclusion, while promoting the implementation of the best practice recommendations on the adoption of telehealth, this project is expected to promote the adoption of telehealth by both healthcare professionals and patients. Such adoption brings implications to the clinical workflow that will move towards the integration of telehealth as a standard complementary practice, thereby enhancing equity in access to telehealth and quality of care at a distance.

## Figures and Tables

**Table 1:** Audit criteria, sample, and audit strategy.

Audit Criteria	Sample	Audit Strategy
1. The healthcare organisation has telehealth policies and procedures.	Quality improvement manager (*n* = 1)	Does the organisation have a telehealth policy/procedure? Compliance: 100% if Yes; 0% if No
2. Patients receive a device to participate in telehealth interventions if needed.	Patients undergoing antineoplastic therapy during the audit period (*n* = 30) and their records	Do you have a device that allows you to participate in telehealth interventions? Do you have an Internet connection through your mobile and/or residency? Compliance: 100% if Yes; 0% if No
3. Healthcare professionals receive training in the use of the applications and devices used for telehealth.	Nurses (*n* = 11), intern physicians (*n* = 9), and oncologists (*n* = 19)	Have you received education and training in telehealth? Compliance: 100% if Yes; 0% if No
4. Healthcare professionals identify potential barriers to telehealth use for each patient.	Patients undergoing antineoplastic therapy during the audit period (*n* = 30) and their records	Have you been asked about potential barriers to participate in telehealth? Compliance: 100% if Yes; 0% if No
5. Healthcare professionals address potential barriers to telehealth use for each patient.	Patients undergoing antineoplastic therapy during the audit period (*n* = 30) and their records	Have any healthcare professional discussed ways of overcoming barriers or challenges to your participation in telehealth? Compliance: 100% if Yes; 0% if No
6. The healthcare organisation outlines costs to patients (if any) for telehealth consultations.	Patients undergoing antineoplastic therapy during the audit period (*n* = 30) and their records	Have you been informed about the cost of participation in telehealth? Compliance: 100% if Yes; 0% if No
7. A dedicated technological support person is available to troubleshoot.	Quality improvement manager (*n* = 1)	Is there a dedicated support person to troubleshoot telehealth issues? Compliance: 100% if Yes; 0% if No

## Data Availability

Not applicable.
